# Comparative analysis of two *Neisseria gonorrhoeae *genome sequences reveals evidence of mobilization of Correia Repeat Enclosed Elements and their role in regulation

**DOI:** 10.1186/1471-2164-10-70

**Published:** 2009-02-09

**Authors:** Lori AS Snyder, Jeff A Cole, Mark J Pallen

**Affiliations:** 1 Systems Biology, University of Birmingham, Edgbaston, Birmingham, UK; 2 School of Biosciences, University of Birmingham, Edgbaston, Birmingham, UK; 3 School of Life Sciences, Kingston University, Penrhyn Road, Kingston upon Thames, KT1 2EE, UK

## Abstract

**Background:**

The Correia Repeat Enclosed Element (CREE) of the *Neisseria *spp., with its inverted repeat and conserved core structure, can generate a promoter sequence at either or both ends, can bind IHF, and can bind RNase III and either be cleaved by it or protected by it. As such, the presence of this element can directly control the expression of adjacent genes. Previous work has shown differences in regulation of gene expression between neisserial strains and species due to the presence of a CREE. These interruptions perhaps remove the expression of CREE-associated genes from ancestral neisserial regulatory networks.

**Results:**

Analysis of the chromosomal locations of the CREE in *Neisseria gonorrhoeae *strain FA1090 and *N. gonorrhoeae *strain NCCP11945 has revealed that most of the over 120 copies of the element are conserved in location between these genome sequences. However, there are some notable exceptions, including differences in the presence and sequence of CREE 5' of copies of the opacity protein gene *opa*, differences in the potential to bind IHF, and differences in the potential to be cleaved by RNase III.

**Conclusion:**

The presence of CREE insertions in one strain relative to the other, CREE within a prophage region, and CREE disrupting coding sequences, provide strong evidence of mobility of this element in *N. gonorrhoeae*. Due to the previously demonstrated role of these elements in altering transcriptional control and the findings from comparing the two gonococcal genome sequences, it is suggested that regulatory differences orchestrated by CREE contribute to the differences between strains and also between the closely related yet clinically distinct species *N. gonorrhoeae*, *Neisseria meningitidis*, and *Neisseria lactamica*.

## Background

**Figure 1:**
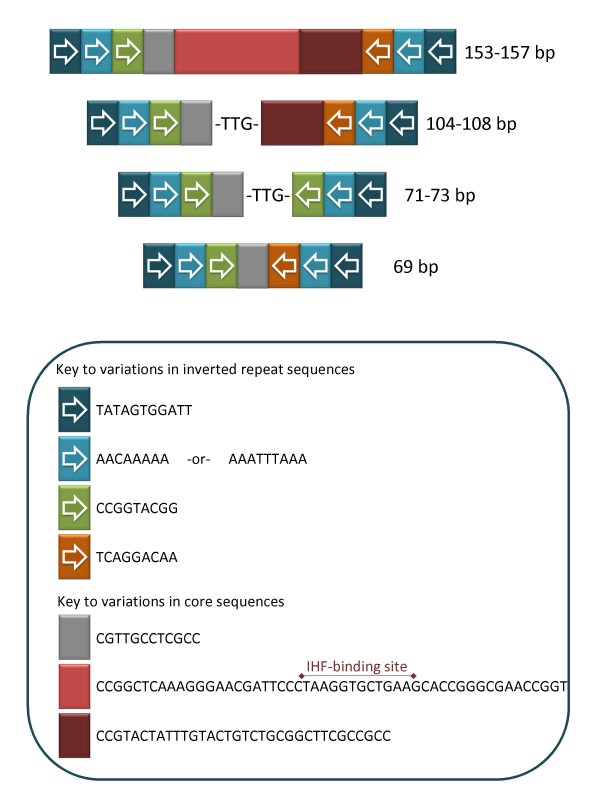
**CREE sequence structures**. Comparative illustration of the sequence structures of different lengths of CREE. Sequences in the key are based on consensus sequences of all aligned *N. gonorrhoeae *CREE. Three different length types of CREE are present in the gonococcal genome sequences, however the shortest (69–73 bp) have two different structure types. An IHF binding site is only possible in the longest of the CREE (153–157 bp).

The genome sequences of the *Neisseria *spp. contain 100 or more copies of a repetitive sequence that has demonstrated roles in gene regulation and is involved in chromosomal changes such as gene inactivation and rearrangements. The Correia Repeat (CR) of 26 bp was first described in 1986 [1], and has only been found in *Neisseria *spp., both pathogenic (*Neisseria gonorrhoeae*, the gonococcus, and *Neisseria meningitidis*, the meningococcus) and commensal (*Neisseria lactamica*) [2,3]. Often it is found as an inverted repeat with a characteristic core [4], the Correia Repeat Enclosed Element (CREE) [5], which is most commonly 153–157 bp or 104–108 bp (Figure 1). The CREE has features of an IS element including the presence of the inverted repeat CR sequences [4] and duplication of the target sequence [6,7]. In addition, there are vast differences in their placement in the neisserial genome sequences and some coding sequences have been disrupted by CREE [3,5,7-9]. This suggests they are, or have been, mobile genetic elements. Unlike IS elements that encode their own mobilizing transposase, the CREE have never been demonstrated to encode proteins and the mobilizing mechanisms for CR and CREE distribution in the chromosome have not been determined.

**Figure 2:**
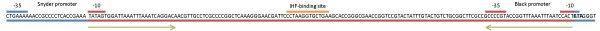
**CREE sequence features**. For illustration purposes a consensus sequence of the longer CREE is shown, here 156 bp. The ways in which the two different CREE associated promoters are generated from native sequence (blue line) and CREE sequence (red line) are shown for the Snyder promoter (left) [11] and the Black promoter (right) [10]. Both of these would drive transcription from left to right. Given favourable native sequence it might be possible to generate both promoters on the opposite strand as well, transcribing right to left. The IHF-binding site within the CREE is shown (orange), as are the inverted repeats that potentially form mRNA hairpin substrates for RNase III (green).

Two functional σ^70 ^promoters have been described as being generated by CREE insertion 5' of genes [10,11] (Figure 2). The first of these, the Black promoter, is at the end of the CREE closest to the gene [10]. The Black promoter is comprised of a -35 and a partial -10 element within the CREE. Insertion in some locations completes the -10 promoter element (Figure 2). Functional Black promoters are present 5' of *uvrB *[10], *drg *[12], *lst *[13], and *mtrCDE *[14]. The second σ^70 ^promoter, the Snyder promoter, was identified at the end of the CREE farthest from the gene. In this case the -35 element comes from the native sequence and the -10 is contained completely within the first 6 bases of the CREE (Figure 2). A functional Snyder promoter was first identified 5' of *dcw *gene *dcaC *in *N. lactamica *[11].

The endoribonuclease RNase III is involved in the processing of stable RNAs such as rRNA and some mRNA transcripts [15]. The stem-loop of the CREE generated by the inverted repeats (Figures 1 and 2) has been determined to be a binding site for RNase III when the CREE sequence is present within the mRNA [16]. The binding of RNase III regulates gene expression post-transcriptionally either through cleavage or protection from cleavage by RNase III, depending on the CR inverted repeat sequence [14,16,17].

The CREE may have other functions as well. For example, some of the longer 153–157 bp CREE contain an IHF binding site (Figures 1 and 2). This may be involved in end synapsis during element transposition [6] or participate in the modulation of regulation of associated genes [14]. CREE may also be hotspots for genomic recombination and rearrangement [5,7,18,19]. Additionally, CREE are associated with gene loss through deletion [18,20] and gene disruption [8,21,22].

Despite all of these known functions, we still do not know whether CREE are currently mobile within these species or whether their mobilization occurred in the distant past in the evolution of these bacteria and the mobilizing element has now been lost. None are present in known or suspected regions of horizontally transferred DNA [6,9,23,24], which suggests that mobility has been lost. However, diversity in the distribution in the *N. meningitidis *sequenced chromosomes [5,8,9,25] and rearrangements between the genome sequences strongly suggest that CREE are mobile in meningococci.

The state of the CREE in *N. gonorrhoeae *could previously only be investigated in the context of its comparison with *N. meningitidis *due to the availability of only one gonococcal genome sequence [GenBank: RefSeq NC_002946]. In the meningococcus over 250 CREE have been identified [5,8,9,25], yet less than half this number have been reported for gonococcal strain FA1090 [5,6]. Is this the result of a species difference in CREE copy number between the gonococcus and the meningococcus? Or, has the relatively longer laboratory propagation of strain FA1090 resulted in the deletion of genetic material including CREE sequences? If the latter is the case then comparison with a more recently acquired and less passaged gonococcal isolate should provide evidence of CREE-mediated deletion events. Likewise, variations in CREE locations between two gonococcal genome sequences might suggest that mobilization has occurred in *N. gonorrhoeae *since its split with the other neisserial species and that such CREE movement might still be occurring. The recent publication of the complete genome sequence of *N. gonorrhoeae *strain NCCP11945, an isolate from a 2002 vaginal smear [26], has opened the way to better understand this human sexually-transmitted pathogen through comparative genome analysis. Here we investigate the CREE of *N. gonorrhoeae *strains FA1090 and NCCP11945.

## Results and discussion

### Identification of CREE in *N. gonorrhoeae *strain FA1090 and *N. gonorrhoeae *strain NCCP11945

**Figure 3:**
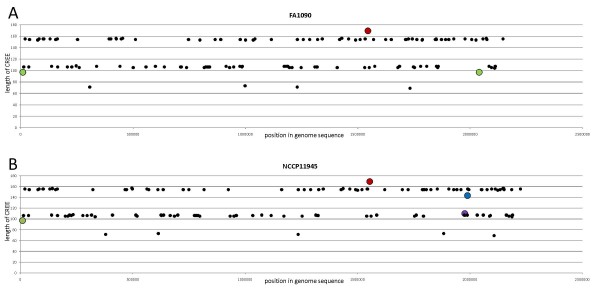
**CREE distribution in the chromosomes**. Distribution of the CREE (x-axis) and their lengths (y-axis) in the genome sequences of *N. gonorrhoeae *strain FA1090 (A) and *N. gonorrhoeae *strain NCCP11945 (B). Length variants are shown with larger spots and are colour coded: green – 97 bp CREE due to a 9 bp deletion; purple – 110 bp CREE due to a 5 bp duplication; blue – 143 bp CREE due to an 11 bp deletion; red – 169 bp CREE due to a 64 bp duplication in a 105 bp CREE.

A new analysis of the *N. gonorrhoeae *strain FA1090 genome sequence was conducted, identifying a total of 123 CREE and 9 single CR sequences (see Additional file 1). A total of 131 CREE and 6 single CR sequences were identified in the genome sequence of *N. gonorrhoeae *strain NCCP11945 (see Additional file 2). The CREE are fairly evenly distributed in the genomes (Figure 3). For both genome sequences, 17 of the CREE contain a perfect IHF binding site based on the published CREE IHF binding site sequence [6], although these 17 are different between the two strains (see Additional file 3). The IHF-binding site-containing CREE are 154 to 157 bp, except for a 143 bp variant that has an 11 bp deletion in its CR in strain NCCP11945 (Figure 3 and Additional file 3). The potential to be cleaved by RNase III is dependent upon the symmetry of the CR ends of the CREE [17]. In strain FA1090, 92 have symmetrical CR ends and 100 CREE have symmetrical ends in strain NCCP11945.

### Most CREE locations are nearly identical in *N. gonorrhoeae *strains FA1090 and NCCP11945

Of the 123 CREE in strain FA1090, 120 are at the same locations in strain NCCP11945 (see Additional file 4). Of these, 83 (69.16%) are nearly identical with 3 or fewer nucleotide differences in sequence and no differences in length. Some of these are 5' of genes that are integral to gonococcal biology (Table 1).

**Table 1:** CREE 5' of important gonococcal genes

**Gene product**	FA1090*	**NCCP11945†**	**CREE distance‡**
Drg restriction endonuclease¶	NGO0007	NGK_0010	22 bp
TonB-dependent receptor	NGO0021	NGK_0029	36 bp
MafB adhesion	NGO0225	NGK_0360	33 bp
UvrB excinuclease subunit**	NGO0573	NGK_1349	124 bp
MarR regulator††	NGO1244	NGK_0263	34 bp
LpxB lipid A biosynthetic pathway protein	NGO1782	NGK_2492	‡‡
HpuA haemoglobin utilization protein	¶¶	NGK_2581	138 bp
Cadmium resistance protein	NGO2127	NGK_2601***	66 bp
RegF pilin regulator	NGO2130	NGK_2604	42 bp
ComE competence protein	NGO1178	NGK_1527	47 bp
	NGO1304	NGK_1966	
	NGO1902	NGK_2096	

* Locus annotation from *N. gonorrhoeae *strain FA1090 [GenBank: RefSeq NC_002946].

† Locus annotation from *N. gonorrhoeae *strain NCCP11945 [GenBank: RefSeq NC_011035].

‡ Distance the CREE is from the initiation codon of the gene.

¶As reported previously [12].

** As reported previously [10].

†† Both types of CREE-associated promoters [10,11] are present. This regulator is specific to the pathogenic *Neisseria *spp. and controls different regulons in *N. gonorrhoeae *and *N. meningitidis *[34].

‡‡ The A of the ATG initiation codon is the final A of the CREE.

¶¶ Unannotated in strain FA1090 where the CDS is frame-shifted at a poly-G tract.

*** This CDS is frame-shifted in strain NCCP11945.

The CREE have become associated with various systems within the gonococcus including those involved in LPS biosynthesis, pilus expression, DNA repair, iron acquisition, adhesion, competence, and pathogen-specific gene regulation. It is possible that variations in the presence of CREE associated with such genes may account for differences in strain behaviour and phenotypes that differ between the gonococcus and the meningococcus. For example, in *N. gonorrhoeae *the MtrCDE efflux pump system, involved in antibiotic resistance [27] and *in vitro *survival [28], is regulated by the repressor MtrR [27] and the activator MtrA [29]. In *N. meningitidis*, a CREE has inserted within the regulatory region for *mtrCDE *and removed the expression of these genes from control by MtrR and MtrA [14]. In this case, the CREE is present between the native promoter [29] and the genes, thus the CREE sequence is part of the mRNA transcript and its cleavage by RNase III has been demonstrated [14]. This CREE also contains an IHF-binding site and its presence in the CREE was shown to have a negative impact on transcription levels [14].

### CREE location differences between the gonococcal strains

Of the CREE identified in the genome sequences of *N. gonorrhoeae *strain FA1090 and *N. gonorrhoeae *strain NCCP11945, thirteen are only present in one of the strains (see Additional files 1, 2, and 4). Four of these are within larger regions of difference. Fragments of CREE are found at three other sites. The remaining six are clear insertions of the element, often at a TA target site as has been described [7]. However, two insertions appear to have occurred at CA sites (strain NCCP11945 positions 1,577,266 and 2,185,647) and indeed some CREE have terminal CA sequences rather than TA (see Additional files 1 and 2). These insertions of CREE in just one of the gonococcal strains support the mobility of the element within the gonococcus and suggest that the mobilization mechanism for the CREE could not have been lost before the speciation event that generated *N. gonorrhoeae *and *N. meningitidis*.

**Figure 4:**
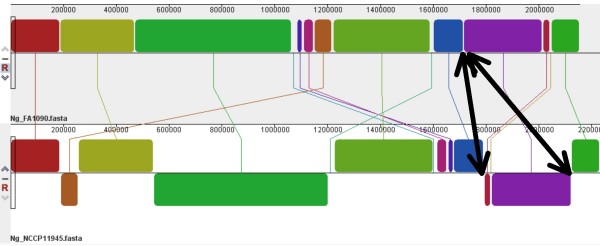
**CREE and chromosomal rearrangement**. Alignment of the *N. gonorrhoeae *strain FA1090 (top) and strain NCCP11945 (bottom) genome sequences using progressive Mauve [43]. Several chromosomal rearrangements and inversions of large regions of sequence can be seen represented here by coloured blocks. Those below the mid-line in strain NCCP11945 are inverted relative to the strain FA1090 sequence. The arrows indicate the left and right ends of a single CREE in strain FA1090 and their alignment with two separate CREE at a chromosomal rearrangement point relative to strain NCCP11945.

Also identified was one CREE in strain FA1090 that corresponded to two different CREE in strain NCCP11945 (Figure 4). The associated chromosomal rearrangement has not created fragments of CREE in either strain.

### *N. gonorrhoeae *have less than half the CREE copies of *N. meningitidis*

It is clear that the gonococci differ from their meningococcal relatives in the number of CREE copies sustained in their genome sequences, with *N. meningitidis *genomes having over 250 [8,9,25]. The commensal *N. lactamica*, a close relative of the pathogenic neisserial species, has been reported to have three times fewer CREE than *N. meningitidis *[2]. A quick search suggests that there are approximately 100 CREE in the *N. lactamica *ST-640 genome sequence (data not presented). Therefore, the commensal and the gonococcus share similar CREE copy numbers.

Coupled with the functions of the CREE, this suggests that *N. gonorrhoeae *and *N. lactamica *retain more of the ancestral *Neisseria *regulatory networks than *N. meningitidis*, where many of these may now be under the transcriptional control of CREE. This is certainly true for the efflux pump system encoded by *mtrCDE*, where both the gonococcal repression [27] and activation [29] systems have been subverted through CREE insertion in the meningococcus [14]. It has been difficult to assign a species-specific gene set to *N. gonorrhoeae*, *N. meningitidis*, and *N. lactamica*, largely due to horizontal exchange between these naturally competent species [30]. The small number of genetic islands identified that were thought to be unique to one species are either present in both pathogenic species or are not present in all strains of the species in which they were originally found. For example, the Gonococcal Genetic Island is not present in all gonococci [31] and was found in strains of *N. meningitidis *[24]. The meningococcal capsule is absent from some *N. meningitidis *strains including some that have caused invasive disease [32,33]. Other Islands of Horizontal Transfer found in *N. meningitidis *are strain-specific [9,25]. Added to this, only six 'virulence' genes present in all pathogenic *Neisseria *genome sequences were absent from the non-pathogen *N. lactamica *[34]. Evidence increasingly supports the idea that regulation rather than gene complement differentiates the species.

### The Correia Repeat of 26 bp is a fragment of CREE sequence

The single CR sequences found in the two gonococcal genome sequences were often associated with a partial core sequence (6 out of 9 and 4 out of 6). In addition, the vast majority of the CR are part of CREE (see Additional files 1 and 2). This suggests that the 26 bp repeats identified by Correia *et al*. [1] are fragments of CREE, having lost the remainder of the sequence through deletion events. Cases of partial CR (<26 bp) are also found in strain FA1090 that correspond to whole CREE 5' of NGK_2270 (*mafA*) and between NGK_2168 and NGK_2169 in strain NCCP11945 (see Additional file 3). The CREE itself should therefore be seen as the functional unit, rather than the chance occurrence of an inverted pair of CR. This is especially evident in light of the conservation of the core sequences (Figure 1).

### Characteristics of the CREE

The gonococcal CREE are evenly distributed in the chromosomes (Figure 3). Most are of the 154–156 bp or 105–107 bp types, however a small number are shorter than these at 69–73 bp. Sequence feature characteristics of the different lengths of CREE illustrated in Figure 1 show a general conservation of CREE structure based on length. Although some length variants shown in Figure 3 were found, in each case these are modifications of one of the basic CREE structures through deletions and tandem repeat duplications. All CREE lengths can have the variations in the inverted repeat end sequences (Figure 1; AACAAAAA or AAATTTAAA) that have been described previously [6]. Symmetry of these inverted repeats generates the potential for RNase III cleavage [17]. The potential for an IHF-binding site is present only in the longer of the CREE (Figure 1). CREE core sequence directionality has a CCGGTACGG end and a TCAGGACAA end (Figure 1). The 71 to 73 bp CREE are the exception to this structure, having two CCGGTACGG ends, yet these still retain a directionality to their core sequence (Figure 1).

### CREE 5' of genes and roles in regulation

A total of 18 potential Black promoters and 15 potential Snyder promoters were found associated with *N. gonorrhoeae *strain FA1090 CREE, with 7 of these CREE containing both (see Additional file 1). While few potential Black and Snyder promoters were identified, there are 76 CREE in strain FA1090 positioned 5' of a gene. This suggests that the presence of the element in this location influences the regulation or expression of these gene(s). It is likely that RNase III has a role in post-transcriptional regulation of these genes. Of these 76 CREE, 57 have symmetrical inverted repeat ends and are therefore potential substrates for RNase III cleavage if the CREE sequence is part of the transcript. Asymmetrical sites are also influenced by RNase III through binding, therefore RNase III is thought to influence the longevity of all mRNAs containing CREE sequence. Seven CREE differ between the strains in their end symmetry, with four of these located 5' of annotated CDSs (NGO0407, NGO0452, NGO1221, and NGO1347). Likewise, nine CREE 5' of CDSs contain IHF-binding sites that may influence the binding and action of other proteins, including RNA polymerase and RNase III.

### CREE associations with copies of the opacity protein gene opa

The Opa proteins of the *Neisseria *spp. are a family of phase variably expressed, antigenically variable outer membrane proteins involved in attachment and invasion of host cells. Multiple copies of the *opa *gene are found in neisserial genomes and variations in their sequences can mediate different interactions with different host cell receptors [35,36]. There are 11 *opa *genes in strain FA1090 [37] and in strain NCCP11945 (Table 2). Most of the strain FA1090 *opa *genes are unannotated and in several cases the strain NCCP11945 annotation has not identified the initiation codon due to the frame-shift generated by the phase variable CTTCT tract within the gene [38]. The majority of the *opa *genes are associated with a 5' CREE, which in some cases contains an IHF-binding site sequence consensus (Table 2).

**Table 2:** The *opa*-associated CREE

NCCP11945*	**FA1090†**	**CREE?‡**	**IHF?¶**
NGK_0096	near NGO0068	No	n/a
NGK_0102	NGO0070	Both	NCCP11945
NGK_0693	near NGO1077	Both	NCCP11945
NGK_0749	near NGO1037	Both	No
NGK_0847	near NGO0950	No	n/a
NGK_1495	near NGO1279	Both	No
NGK_1729	near NGO1465	Both	Both
NGK_1799	NGO1513	NCCP11945	No
NGK_1847	near NGO1556	Both	No -short
NGK_2170	near NGO2060	No	n/a
NGK_2410	near NGO1861	No	n/a

* Locus annotation from *N. gonorrhoeae *strain NCCP11945 [GenBank: RefSeq NC_011035].

† Locus annotation from *N. gonorrhoeae *strain FA1090 [GenBank: RefSeq NC_002946].

‡ CREE presence.

¶IHF-binding site presence based on 100% identity with the published sequence [6].

In all cases, there appears to be a σ^70 ^promoter between the CREE and *opa*, which would mean that the CREE sequence is not part of the mRNA transcript and that it is not therefore targeted by RNase III. There are sequence differences in the IHF-binding sites between the strains, but whether the sites that differ from the published IHF-binding site sequence are still able to bind IHF is not known. The CREE 5' of NGK_1799 in strain NCCP11945 is not present in strain FA1090, where a 17 bp fragment of the end of the CREE remains 5' of NGO1513. The CREE 5' of NGK_1847 (unannotated in strain FA1090) is only 105 bp and therefore does not carry the IHF-binding site (Figure 1).

Indeed, there are no CREE at all present 5' of the *opa *copies NGK_0096, NGK_0847, NGK_2170, and NGK_2410 (all unannotated in strain FA1090) (Table 2). The regions upstream of these genes are otherwise similar, which would suggest that the CREE was mobile after the gene duplication events that generated 11 copies of *opa*, three to four times the number of *opa *genes found in *N. meningitidis *[8,9,25]. Indeed all of the copies of *opa *found in *N. meningitidis *strain Z2491 have a CREE 98–99 bp 5' (NMA1676, NMA1890, NMA2043), with similarly placed CREE for three out of four *opa *genes in meningococcal strain MC58 (NMB0442, NMB1465, NMB1636, but not NMB0926) [8,9,34]. When the phase variable tract is ON in *opa *the influences of the various upstream sequences, including the CREE, may have further regulatory effects on expression of this important adhesin, perhaps through binding of IHF.

### CREE as insertional inactivators of genes

Of the 5 previously reported genes disrupted by CREE insertion [8,21,22], 4 are associated with CREE in these two gonococcal genome sequences (Table 3). In the case of NMA2121, this CDS is not present in strain FA1090 as it is part of a Minimal Mobile Element [22,39,40], which facilitates horizontal exchange of gene cassettes between genome sequences.

**Table 3:** Disruption of annotated coding regions by CREE

	FA1090*	**CREE?†**	**NCCP11945‡**	**CREE?†**
**previously reported:**				
NMA2121	not present	n/a	NGK_1900	Yes
NMB0397	NGO1564**	no	NGK_1865/7	Yes
*cvaB*	unannotated††	yes	NGK_0171	Yes
NMA0059	unannotated	yes	NGK_2501	Yes
NMA0530	NGO2162	no	NGK_2641	No
**new from this study:**				
	NGO0243	yes	NGK_0377	Yes
	NGO2104	yes	NGK_2573	Yes
	NGO1597	yes	NGK_1901‡‡	No

* Locus annotation from *N. gonorrhoeae *strain FA1090 [GenBank: RefSeq NC_002946].

† Presence of a CREE.

‡ Locus annotation from *N. gonorrhoeae *strain NCCP11945 [GenBank: RefSeq NC_011035].

¶Differences in annotation place this CREE 21 bp 5' of NGK_1900 rather than disrupting the CDS.

** This CDS disrupts the NMB0397 orthologue in this strain.

†† The corresponding sequence in this strain has a 954 bp deletion relative to NGK_0171.

‡‡ This CDS is annotated on the reverse complement strand from NGO1597. By this annotation the site of CREE insertion would be 3' of the CDS.

Three additional coding sequence disruptions by CREE were identified in strains FA1090 and NCCP11945 (Table 3). In two of these, the disruption is shared by both strains, while the third has no CREE in this region in strain NCCP11945. The fact that the CREE has inserted into identifiable coding regions supports the hypothesis that this element is, or was, mobile. That disruption of some CDSs is seen only in the gonococcus supports mobilization of the CREE within *N. gonorrhoeae *since its split from *N. meningitidis*.

### CREE within potential regions of horizontal transfer

It has been proposed that CREE are not found in regions of horizontal transfer and that their absence can be taken as additional evidence for the horizontal origin of a region, given their otherwise even distribution in the chromosome [6,9,23,24]. The CREE sequences 38 bp 5' of NGO1004, 213 bp 5' of NGO1006, and 21 bp 3' of NGO1020, present in both gonococcal strains, are within a 13 kb region between *rpoD *and tRNA-Arg containing 17 CDSs annotated as "putative phage associated proteins". This demonstrates that the CREE has been mobile at some time since the acquisition of this prophage sequence in the *Neisseria*. While it might be possible to use the absence of CREE as additional evidence indicating foreign sequence origin, as for the meningococcal IHTs [9] and the Gonococcal Genetic Island [24], the presence of CREE does not preclude the possibility of horizontal transfer of the region.

## Conclusion

The CREE are less numerous in the gonococcal genome sequences (~120 copies) than in the meningococcal genome sequences (~250 copies). Many of the gonococcal CREE (71 of 113 in strain FA1090) are 5' of genes and in this position are likely to be involved in the regulation of genes through generation of promoter sequences, binding of IHF, and mRNA stability control by RNase III. CREE are associated with virulence- and host survival-associated determinants such as Opa, HpuA, and MafB, as well as regulators of factors important in pathogenesis. Differences in CREE insertions between the two strains and the presence of CREE within a prophage region, upstream of multiple copies of *opa*, and within coding sequences provide strong evidence of mobility of this element in the gonococcus and therefore since the speciation event that differentiated the gonococcus from the meningococcus. The regulatory influence of the CREE and the copy number differences between *N. gonorrhoeae*, *N. meningitidis*, and *N. lactamica *may contribute to the different behaviours of these pathogens through differences in their regulatory networks.

## Methods

### CREE identification in the genome sequences

The genome sequences of *N. gonorrhoeae *strain FA1090 [GenBank: RefSeq NC_002946] and *N. gonorrhoeae *strain NCCP11945 [GenBank: RefSeq NC_011035] were searched for CR sequences using the tools within *x*BASE [41]. Each genome sequence was searched using 'fuzznuc' for three different patterns based on the conserved CR sequences identified from previous studies of this element [5,6,8,25]: TATAG [CT]GGATTAACAAAAATCAGGAC; TATAG [CT]GGATTAAATTTAAACCGGTAC; TATAG [CT]GGATTAACAAAAACCGGTAC; and TATAG [CT]GGATTAAATTTAAATCAGGAC. Square brackets will find either of the letters within them. In each case, up to 3 mismatches were allowed. Increasing to 4 or 5 mismatches did not identify any additional CREE. Pattern finding has been successfully used previously to identify CREE [3,5]. Each identified location was then investigated manually to determine if the site contained a lone CR or a CREE. Each location was catalogued and the length and sequence of the CREE was determined (see Additional files 1 and 2). As a confirmation, searches with the above sequences were also conducted using BLASTN, as has been done previously [5-7], against the gonococcal genome sequences and also against *N. lactamica *ST-640 (data not presented). IHF sites were found by searching within the CREE sequences for the sequence CTTCAGCACCTTA [6] (see Additional file 3). RNase III cleavage potential was determined based on CR sequence and symmetry between the ends of the CREE.

### Genome sequence comparative analysis

Using *x*BASE [41], WebACT [42], and progressive Mauve [43], the genome sequence data from gonococcal strains FA1090 and NCCP11945 were comparatively aligned and visualized. For each CREE location the presence of a corresponding CREE in the other genome sequence, if present, was catalogued (see Additional file 4).

### CREE sequence alignment

For each site where CREE were found in both the gonococcal genome sequences, the sequences of the CREE were aligned using EMBOSS needle [44] and the calculated percent identity was recorded (see Additional file 4). All of the identified CREE sequences were aligned together using ClustalW2 [45] from EMBL-EBI.

## Other nomenclature used

### Full length inverted repeats

Correia repeats; Correia elements (CE); Correia; Correia full (~150 bp); Correia internal deletion (~105 bp); *Neisseria *miniature insertion sequences (*nemis*); neisserial repetitive sequences; pseudo-transposable neisserial small repetitive elements (SRE); *N. gonorrhoeae *inverted repeats; Correia sequences; 152-bp repetitive sequence.

### Single repeats

Correia repeats; Correia repeat unit; 26-bp repeat; 26-bp neisserial repeat (NR).

## Authors' contributions

LS conceived of the study, carried out the comparative genome analyses, data collection, and drafted the manuscript. JC and MP assisted in the interpretation of the data and suggested improvements to the presentation of the results and their discussion in the manuscript. All authors read and approved the final manuscript.

## Supplementary Material

Additional File 1 **CREE in *N. gonorrhoeae *strain FA1090.** This spreadsheet contains the locations, sequence, and features of all of the CREE identified in the genome sequence of *N. gonorrhoeae *strain FA1090. Click here for file

Additional File 2 **CREE in *N. gonorrhoeae *strain NCCP11945.** This spreadsheet contains the locations, sequence, and features of all of the CREE identified in the genome sequence of *N. gonorrhoeae *strain NCCP11945. Click here for file

Additional File 3 **Comparison of long CREE and IHF-binding sites between *N. gonorrhoeae *strains FA1090 and NCCP11945.** This spreadsheet contains comparative data on the long CREE in *N. gonorrhoeae *strain FA1090 and *N. gonorrhoeae *strain NCCP11945 and the presence of an IHF-binding site that corresponds to the sequence published [6]. Click here for file

Additional File 4 **Comparison of CREE locations between *N. gonorrhoeae *strains FA1090 and NCCP11945.** This spreadsheet contains the CREE that are in common between *N. gonorrhoeae *strain FA1090 and *N. gonorrhoeae *strain NCCP11945. Click here for file

## References

[B1] Correia FF, Inouye S, Inouye M (1986). A 26-base-pair repetitivesequence specific for *Neisseria gonorrhoeae *and *Neisseria meningitidis *genomic DNA. J Bacteriol.

[B2] De Gregorio E, Abrescia C, Carlomagno MS, Di Nocera PP (2003). Asymmetrical distribution of *Neisseria *miniature insertion sequence DNA repeats among pathogenic and nonpathogenic *Neisseria *strains. Infect Immun.

[B3] Qvarnstrom Y, Swedberg G (2006). Variations in gene organization and DNA uptake signal sequence in the *folP *region between commensal and pathogenic *Neisseria *species. BMC Microbiol.

[B4] Correia FF, Inouye S, Inouye M (1988). A family of small repeated elements with some transposon-like properties in the genome of *Neisseria gonorrhoeae*. J Biol Chem.

[B5] Liu SV, Saunders NJ, Jeffries A, Rest RF (2002). Genome analysis and strain comparison of correia repeats and correia repeat-enclosed elements in pathogenic *Neisseria*. J Bacteriol.

[B6] Buisine N, Tang CM, Chalmers R (2002). Transposon-like Correia elements: structure, distribution and genetic exchange between pathogenic *Neisseria *sp. FEBS Lett.

[B7] Mazzone M, De Gregorio E, Lavitola A, Pagliarulo C, Alifano P, DiNocera PP (2001). Whole-genome organization and functional properties of miniature DNA insertion sequences conserved in pathogenic Neisseriae. Gene.

[B8] Parkhill J, Achtman M, James KD, Bentley SD, Churcher C, Klee SR, Morelli G, Basham D, Brown D, Chillingworth T (2000). Complete DNA sequence of a serogroup A strain of *Neisseria meningitidis *Z2491. Nature.

[B9] Tettelin H, Saunders NJ, Heidelberg J, Jeffries AC, Nelson KE, Eisen JA, Ketchum KA, Hood DW, Peden JF, Dodson RJ (2000). Complete genome sequence of *Neisseria meningitidis *serogroup B strain MC58. Science.

[B10] Black CG, Fyfe JA, Davies JK (1995). A promoter associated with the neisserial repeat can be used to transcribe the *uvrB *gene from *Neisseria gonorrhoeae*. J Bacteriol.

[B11] Snyder LA, Shafer WM, Saunders NJ (2003). Divergence and transcriptional analysis of the division cell wall (*dcw*) gene cluster in *Neisseria *spp. Mol Microbiol.

[B12] Cantalupo G, Bucci C, Salvatore P, Pagliarulo C, Roberti V, Lavitola A, Bruni CB, Alifano P (2001). Evolution and function of the neisserial *dam*-replacing gene. FEBS Lett.

[B13] Packiam M, Shell DM, Liu SV, Liu YB, McGee DJ, Srivastava R, Seal S, Rest RF (2006). Differential expression and transcriptional analysis of the alpha-2,3-sialyltransferase gene in pathogenic *Neisseria *spp. Infect Immun.

[B14] Rouquette-Loughlin CE, Balthazar JT, Hill SA, Shafer WM (2004). Modulation of the *mtrCDE*-encoded efflux pump gene complex of *Neisseria meningitidis *due to a Correia element insertion sequence. Mol Microbiol.

[B15] Nikolaev N, Silengo L, Schlessinger D (1973). A role for ribonuclease 3 in processing of ribosomal ribonucleic acid and messenger ribonucleic acid precursors in *Escherichia coli*. J Biol Chem.

[B16] De Gregorio E, Abrescia C, Carlomagno MS, Di Nocera PP (2002). The abundant class of nemis repeats provides RNA substrates for ribonuclease III in Neisseriae. Biochim Biophys Acta.

[B17] De Gregorio E, Abrescia C, Carlomagno MS, Di Nocera PP (2003). Ribonuclease III-mediated processing of specific *Neisseria meningitidis *mRNAs. Biochem J.

[B18] Claus H, Elias J, Meinhardt C, Frosch M, Vogel U (2007). Deletion of the meningococcal *fetA *gene used for antigen sequence typing of invasive and commensal isolates from Germany: frequencies and mechanisms. J Clin Microbiol.

[B19] Davidsen T, Tonjum T (2006). Meningococcal genome dynamics. Nat Rev Microbiol.

[B20] Ende A van der, Hopman CT, Dankert J (1999). Deletion of *porA *by recombination between clusters of repetitive extragenic palindromic sequences in *Neisseria meningitidis*. Infect Immun.

[B21] Klee SR, Nassif X, Kusecek B, Merker P, Beretti JL, Achtman M, Tinsley CR (2000). Molecular and biological analysis of eight genetic islands that distinguish *Neisseria meningitidis *from the closely related pathogen *Neisseria gonorrhoeae*. Infect Immun.

[B22] Snyder LA, Davies JK, Saunders NJ (2004). Microarray genomotyping of key experimental strains of *Neisseria gonorrhoeae *reveals gene complement diversity and five new neisserial genes associated with Minimal Mobile Elements. BMC Genomics.

[B23] Snyder LA, Davies JK, Ryan CS, Saunders NJ (2005). Comparativeoverview of the genomic and genetic differences between the pathogenic *Neisseria *strains and species. Plasmid.

[B24] Snyder LA, Jarvis SA, Saunders NJ (2005). Complete and variant forms of the 'gonococcal genetic island' in *Neisseria meningitidis*. Microbiology.

[B25] Bentley SD, Vernikos GS, Snyder LA, Churcher C, Arrowsmith C, Chillingworth T, Cronin A, Davis PH, Holroyd NE, Jagels K (2007). Meningococcal genetic variation mechanisms viewed through comparative analysis of serogroup C strain FAM18. PLoS Genet.

[B26] Chung GT, Yoo JS, Oh HB, Lee YS, Cha SH, Kim SJ, Yoo CK (2008). Complete genome sequence of *Neisseria gonorrhoeae *NCCP11945. J Bacteriol.

[B27] Hagman KE, Pan W, Spratt BG, Balthazar JT, Judd RC, Shafer WM (1995). Resistance of *Neisseria gonorrhoeae *to antimicrobial hydrophobic agents is modulated by the *mtrRCDE *efflux system. Microbiology.

[B28] Jerse AE, Sharma ND, Simms AN, Crow ET, Snyder LA, Shafer WM (2003). A gonococcal efflux pump system enhances bacterial survival in a female mouse model of genital tract infection. Infect Immun.

[B29] Rouquette C, Harmon JB, Shafer WM (1999). Induction of the *mtrCDE*-encoded efflux pump system of *Neisseria gonorrhoeae *requires MtrA, an AraC-like protein. Mol Microbiol.

[B30] Maiden MC (2008). Population genomics: diversity and virulence in the *Neisseria*. Curr Opin Microbiol.

[B31] Dillard JP, Seifert HS (2001). A variable genetic island specific for *Neisseria gonorrhoeae *is involved in providing DNA for natural transformation and is found more often in disseminated infection isolates. Mol Microbiol.

[B32] Findlow H, Vogel U, Mueller JE, Curry A, Njanpop-Lafourcade BM, Claus H, Gray SJ, Yaro S, Traore Y, Sangare L (2007). Three cases of invasive meningococcal disease caused by a capsule null locus strain circulating among healthy carriers in Burkina Faso. J Infect Dis.

[B33] Hoang LM, Thomas E, Tyler S, Pollard AJ, Stephens G, Gustafson L, McNabb A, Pocock I, Tsang R, Tan R (2005). Rapid and fatal meningococcal disease due to a strain of *Neisseria meningitidis *containing the capsule null locus. Clin Infect Dis.

[B34] Snyder LA, Saunders NJ (2006). The majority of genes in the pathogenic *Neisseria *species are present in non-pathogenic *Neisseria lactamica*, including those designated as 'virulence genes'. BMC Genomics.

[B35] Hauck CR, Meyer TF (2003). 'Small' talk: Opa proteins as mediators of *Neisseria*-host-cell communication. Curr Opin Microbiol.

[B36] Gray-Owen SD (2003). Neisserial Opa proteins: impact on colonization, dissemination and immunity. Scand J Infect Dis.

[B37] Snyder LA, Butcher SA, Saunders NJ (2001). Comparative whole-genome analyses reveal over 100 putative phase-variable genes in the pathogenic *Neisseria *spp. Microbiology.

[B38] Muralidharan K, Stern A, Meyer TF (1987). The control mechanism of opacity protein expression in the pathogenic Neisseriae. Antonie Van Leeuwenhoek.

[B39] Saunders NJ, Snyder LA (2002). The minimal mobile element. Microbiology.

[B40] Snyder LA, McGowan S, Rogers M, Duro E, O'Farrell E, Saunders NJ (2007). The repertoire of minimal mobile elements in the *Neisseria *species and evidence that these are involved in horizontal gene transfer in other bacteria. Mol Biol Evol.

[B41] Chaudhuri RR, Loman NJ, Snyder LA, Bailey CM, Stekel DJ, Pallen MJ (2008). *x*BASE2: a comprehensive resource for comparative bacterial genomics. Nucleic Acids Res.

[B42] Carver TJ, Rutherford KM, Berriman M, Rajandream MA, Barrell BG, Parkhill J (2005). ACT: the Artemis Comparison Tool. Bioinformatics.

[B43] Darling AC, Mau B, Blattner FR, Perna NT (2004). Mauve: multiple alignment of conserved genomic sequence with rearrangements. Genome Res.

[B44] Needleman SB, Wunsch CD (1970). A general method applicable to the search for similarities in the amino acid sequence of two proteins. J Mol Biol.

[B45] Larkin MA, Blackshields G, Brown NP, Chenna R, McGettigan PA, McWilliam H, Valentin F, Wallace IM, Wilm A, Lopez R (2007). Clustal W and Clustal X version 2.0. Bioinformatics.

